# Pattern detection in the vehicular activity of bus rapid transit systems

**DOI:** 10.1371/journal.pone.0312541

**Published:** 2024-10-29

**Authors:** Jaspe U. Martínez-González, Alejandro P. Riascos, José L. Mateos

**Affiliations:** 1 Instituto de Física, Universidad Nacional Autónoma de México, Ciudad Universitaria, Ciudad de México, México; 2 Departamento de Física, Universidad Nacional de Colombia, Bogotá, Colombia; 3 Centro de Ciencias de la Complejidad, Universidad Nacional Autónoma de México, Ciudad de México, México; SASTRA Deemed University: Shanmugha Arts Science Technology and Research Academy, INDIA

## Abstract

In this paper, we explore different methods to detect patterns in the activity of bus rapid transit (BRT) systems focusing on two aspects of transit: infrastructure and the movement of vehicles. To this end, we analyze records of velocity and position of each active vehicle in nine BRT systems located in the Americas. We detect collective patterns that characterize each BRT system obtained from the statistical analysis of velocities in the entire system (global scale) and at specific zones (local scale). We analyze the velocity records at the local scale applying the Kullback-Leibler divergence to compare the vehicular activity between zones. This information is organized in a similarity matrix that can be represented as a network of zones. The resulting structure for each system is analyzed using network science methods. In particular, by implementing community detection algorithms on networks, we obtain different groups of zones characterized by similarities in the movement of vehicles. Our findings show that the representation of the dataset with information of vehicles as a network is a useful tool to characterize at different scales the activity of BRT systems when geolocalized records of vehicular movement are available. This general approach can be implemented in the analysis of other public transportation systems.

## Introduction

In the last decades, the study of cities has emerged from diverse scientific disciplines [1–5]. This integration is facilitated by the understanding of cities as complex systems [6, 7], offering a rich framework for the application of mathematical methods in urban studies [1, 3]. Furthermore, the recent availability of data records for diverse aspects of daily urban life has enabled the detailed characterization of human movement, allowing the identification of behavioral patterns at different scales [8–13]. In particular, one crucial aspect of urban dynamics is the public transport, which is intertwined with key urban issues such as optimization of the existing infrastructure [14, 15], disease spread [16], social disparities [17, 18] and economic inequalities [19], segregation [20], among many others [1, 3, 7].

The complexity of urban mobility constitutes a challenge. However, when elements of this complexity are represented as a network, it becomes more feasible to discern its properties. This approach has been proven effective in studying various other complex systems [21, 22]. For instance, by abstracting mobility infrastructure as a network [23–25], it is possible to characterize the physical connectivity of these systems. Similarly, the representation of human activity datasets as a network [26, 27] allows to delve into deeper properties at different scales. In this manner, the methods of network science serve as a valuable bridge between available data and recently developed physical models, incorporating concepts such as phase transitions and percolation [28–30]. Furthermore, other network properties, like community structure, can reveal patterns in vehicular activity at different scales [11].

Building on these insights, we implement different tools of network science to analyze nine bus rapid transit (BRT) systems. The term BRT refers to a bus-based system, where vehicles transit over exclusive lanes. Also, fulfill other characteristics, like the infrastructure of stops that include entering systems based on electronic cards or a schedule on arrival times to the stops [31, 32]. BRT systems appeared in the 1970 decade [31, 33] and, nowadays almost 200 systems exist worldwide [32]. This urban transit system is considered an alternative to other transport infrastructure like metro or light rail, due to their lesser costs of construction, operation, and maintenance [33, 34], meeting the transportation needs of urban residents [35]. Our focus on BRT systems is motivated by the reduced number of prior studies investigating its network infrastructure and the characterization of vehicular activity.

In this research, we explore BRT systems using network science in two aspects. First, we examine the networks associated with the infrastructure of these systems to determine features related to their connectivity. In the second part, we explore networks associated with vehicular activity within the systems. For the analysis of the movement of vehicles; initially, we consider 135 468 825 data records including a timestamp, position, and velocity of each active vehicle in nine BRT systems. Using this information we track vehicular activity at both the global level, covering the entire system, and at a local level within specific zones. For the latter, we divided the system into polygons considering the position of stations. Different statistical methods including the Kullback-Leibler divergence are implemented to compare the vehicular activity between every pair of polygons in each system. The outcomes are organized into similarity matrices that provide information to generate networks where each node is a segment of the system and links are associated with similar activity of vehicles at the local level. We apply a community detection algorithm to identify the community structure within similarity networks; this particular organization of the network allows a multiscale characterization of the movement of vehicles in the systems explored. The results show that the methods of network science are an important tool to characterize the activity of BRT systems when geolocalized records of vehicular movement are available. The approach explored is general and can be useful in the study of other public transportation systems.

## Materials and methods

### Data description

In this section, we describe the datasets of BRT systems considered in our research. We start with the information in two websites, [36, 37], and other sources on the internet to collect some links to useful databases. We explore 187 BRT systems worldwide detected with the implementation of the following procedure. First, we identify cities having open access to the data of transportation systems; the protocol of the required data is called “Real Time” because the information is actualized within a certain period to have current datasets of the transportation systems. In this information, we check if the available data contains records of the bus rapid transit system. Then, once identified the data it is necessary to check if the geographical coordinates (latitude and longitude) and the velocity of each active vehicle in the system are included in the available records. From this exploration, we determine nine BRT systems in the Americas providing the information to be analyzed. The BRT systems considered in our research are located in the cities of Louisville (Kentucky, USA), Austin (Texas, USA), Nashville (Tennessee, USA), San Antonio (Texas, USA), Maui (Hawaii, USA), Brampton, (Ontario, Canada), Rio de Janeiro (Brazil), Hartford (Connecticut, USA) and Mexico City (Mexico). The data collection and analysis in this study comply with the terms and conditions of the data source.

We download the data from each official source, considering their particular operational characteristics including the refresh time of the data packages and the service schedule. The datasets were processed in a human-readable format and saved in comma-separated value files at the end of each day. The information was downloaded from 2022 May 20th to 2023 May 19th. One particular case is the BRT system in Rio de Janeiro, whose datasets were downloaded from a project of Google Cloud where the authorities of the city store its open data [38], the records include the activity of vehicles from 2022 May 1st to 2023 April 30th.

In Table 1, we summarize the information considered in this research for nine BRT systems. We present the city where the system is located, the name of the BRT system as well as the link where the data was obtained. We also include the number of days in which it was possible to download at least one record of the geographic coordinates, along with the velocity *v* of vehicles in the system for which 0 km/h < *v* ≤ 90 km/h and the total number of these records. The last column shows the total number of active vehicles for which we have at least one record over the download period. The values presented in the table give an idea of the massive amount of data that is available when considering the global activity of a BRT system. The continuous monitoring of vehicles presents a challenge for the analysis of this information. In this research, several methods for studying global vehicle activity as well as in specific zones of the city are explored. This approach allows to classify and detect patterns of the movement of vehicles at different scales.

**Table 1:** Datasets considered for nine BRT systems. Information about the city where each system is located, the name of the system, the link where the data is available, the number of days considered in the dataset, the total number of records with velocities 0 km/h < *v* ≤ 90 km/h and the total number of active vehicles.

City	System Name	Days	Number of records	Active vehicles
Louisville	Dixie Rapid [39]	335	977397	109
Austin	Metro Rapid [40]	345	10620212	711
Nashville	BRT Lite [41]	344	10144734	207
San Antonio	VIA Prímo [42]	289	3224836	506
Maui	Maui Bus [43]	273	4403523	41
Brampton	Züm [44]	327	1730986	38
Rio de Janeiro	BRT Río [38]	348	24775302	777
Hartford	CT Fastrak [45]	338	6993376	248
Mexico City	Metrobús [46]	218	72598459	899

### Infrastructure networks

**Fig 1:**
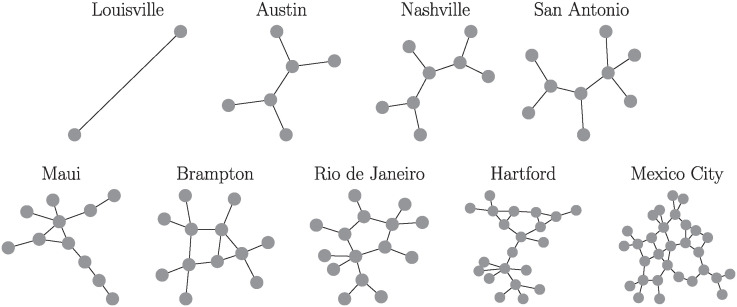
Infrastructure networks of nine BRT systems. Network representation of BRT systems considered in this research sorted by the number of nodes.

An important characteristic of transportation networks is their connectivity features, due to the need in urban transport to reach as many points of interest as possible. A first approach to understanding the connectivity of BRT systems is to focus on end and transfer stations. Transfer stations show how lines of the systems are connected while end stations determine the direction that passengers take once they enter the system. Based on this point of view it is possible to represent a transportation system as an undirected graph, where the end and transfer stations are nodes, and the edges are the paths between them; we refer as *infrastructure network* to this graph describing a BRT system. This particular representation was introduced by Derrible for the analysis of metro systems [23]. In Fig 1, we show the infrastructure networks obtained for the nine BRT systems considered in this research.

The representation of BRT systems as networks allows a first analysis of their infrastructure with different well-known quantities and properties studied by network science [22]. The first two quantities that describe the systems are the number of nodes *N* and the number of edges |E|. Furthermore, an essential quantity in the study of a network is the number of nodes connected to a node *i*, this is the degree *k*_*i*_. In infrastructure networks, a higher degree *k*_*i*_ reveals that several lines intersect at the station *i*. A global view of this feature on undirected networks is given by the average degree 〈*k*〉 that satisfies $\langle k\rangle \equiv \frac{1}{N}\sum_{i=1}^{N}k_{i}=2\left|E\right|/N$. In addition, we can characterize how well-connected a transportation system is by counting the number of changes between lines a user have to make in order to reach a particular destination. If the number of changes is low, the system approaches an optimal case. This feature is represented in its graph by the number of edges *l*_*ij*_ of the shortest path connecting a pair of nodes *i*, *j*. Globally, the entire network is characterized by the average length of the shortest path 〈*l*〉, defined by [47]

$$\begin{array}{c} \left\langle l\right\rangle \equiv \frac{1}{N(N-1)}\sum_{i,j=1}^{N}l_{ij}. \end{array}$$ (1)

Another common quantity that describes the structure of a network is the average clustering coefficient 〈*C*〉, defined as [47]

$$\begin{array}{c} \left\langle C\right\rangle \equiv \frac{1}{N}\sum_{i=1}^{N}C_{i}, \end{array}$$ (2)

where *C*_*i*_ = 0 if *k*_*i*_ = 1 and

$$\begin{array}{c} C_{i}=\frac{2\times \text{number}\;\text{of}\;\text{triangles}\;\text{around}\;\text{node}\;i}{k_{i}\left(k_{i}-1\right)}, \end{array}$$ (3)

for *k*_*i*_ ≥ 2. Then, 〈*C*〉 is a measure that gives the proportion of triangles in the network.

Once described some common quantities to analyze networks, let us apply these measures to the infrastructure networks shown in Fig 1. Our findings are reported in Table 2. In the first three columns, we present general information: the city where the BRT system is located, the number of lines and stations in the data download period. The next five columns include information about the infrastructure networks describing each system. We show the number of nodes *N*, the total number of edges |E|, the average degree 〈*k*〉, the average length of the shortest path 〈*l*〉 and the average clustering coefficient 〈*C*〉. All the information reported in Table 2 is sorted using the number of nodes *N* of the infrastructure networks from small BRT systems such as Louisville’s Dixie Rapid to larger ones such as Mexico City’s Metrobus system. In particular, the BRT in Louisville has only two nodes, because the system is composed of one line, and hence, only the end stations are considered as nodes.

**Table 2:** Properties of the infrastructure networks of nine BRT systems. Information about the city where each system is located, the number of lines, and the number of stations. Also are included the size of the infrastructure network *N*, the number of edges |E|, the average degree 〈*k*〉, the average length of the shortest path 〈*l*〉 as defined in Eq (1) and the average clustering coefficient 〈*C*〉 in Eq (2).

City	Lines	Stations	*N*	|E|	〈*k*〉	〈*l*〉	〈*C*〉
Louisville	1	16	2	1	1.0	1.0	0.0
Austin	2	52	6	5	1.67	1.93	0.0
Nashville	4	172	8	7	1.75	2.32	0.0
San Antonio	3	75	9	8	1.78	2.39	0.0
Maui	12	217	11	11	2.0	2.69	0.0697
Brampton	4	97	12	13	2.17	2.42	0.0556
Rio de Janeiro	3	137	15	15	2.0	2.76	0.0
Hartford	8	265	21	23	2.19	3.62	0.0873
Mexico City	7	283	30	37	2.47	3.92	0.0444

Moreover, regarding the average degrees 〈*k*〉, their values lie in the range 1 ≤ 〈*k*〉 < 3. This result shows a particular characteristic of infrastructure networks that contrasts with complex networks found in the study of human mobility (for example in taxis [27], bike sharing systems [26] or airports [48]). In addition, the global connectivity of a transportation system is characterized by counting the number of changes between lines of users to reach a particular destination. If the number of changes is low, the system approaches an ideal case. This feature of a network is quantified by the average shortest path and the results show that 1 ≤ 〈*l*〉 < 4. Therefore, in the BRT systems explored, the passengers need on average a low number of transfers between lines to reach a destination. Finally, concerning the average clustering coefficient 〈*C*〉, our findings show that most of the systems have null values. For example, in Fig 1, the infrastructure networks associated with Louisville, Austin, Nashville, and San Antonio are graphs with no cycles, then 〈*C*〉 = 0. In the case of the system in Rio de Janeiro, the structure has one cycle with five nodes also producing 〈*C*〉 = 0. Only Brampton, Hartford, Maui, and Mexico City have nodes that form triangles within the network. In general, the existence of cycles with different sizes reveals alternative routes that the passenger can choose in order to reach the final station. This is an important feature to consider in urban transportation systems; a recent work for networks of metro systems has shown that the robustness of infrastructure networks increases when multiple paths in the system can connect two nodes [49].

### Global activity of vehicles in BRT systems

In addition to the infrastructure of BRT systems, it is important to characterize the movement of vehicles. Due to their specific features: exclusive lanes, velocity limits, and stops at determined bus stations, it is crucial to understand if these particularities in the vehicular activity lead to the emergence of patterns at different scales in urban areas. In this section, we analyze statistically the information of velocities considering all the active vehicles in the nine BRT systems described in Table 1, for which we have the geographical coordinates and the speed of all the vehicles. The analysis of the velocity of vehicles accumulated over a certain period gives information about behavior patterns over the entire system.

**Fig 2:**
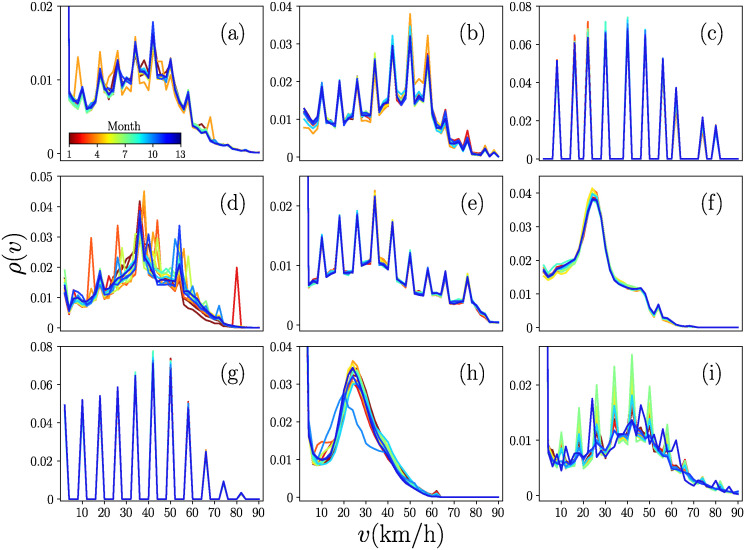
Monthly probability densities *ρ*(*v*) of velocities *v* at the global level. Monthly probability densities *ρ*(*v*) as a function of the velocity *v* in the systems in (a) Austin, (b) Brampton, (c) Hartford, (d) Louisville, (e) Maui, (f) Mexico City, (g) Nashville, (h) Rio de Janeiro and (i) San Antonio. Each *ρ*(*v*) is colored according to the month analyzed from May 2022 to May 2023 (13 months codified in the colorbar in panel (a)). The values of *ρ*(*v*) are obtained using bin counts in the interval 0 km/h < *v* ≤ 90 km/h with size Δ*v* = 2 km/h.

In Fig 2, we present the probability density *ρ*(*v*) of the velocities *v* for the BRT systems for each month covered by our study. The nine systems are sorted alphabetically using the name of the cities where they are located, starting from Austin and ending with San Antonio. All the results for *ρ*(*v*) are obtained using bin counts with size Δ*v* = 2 km/h, this bin size is maintained for all the systems. The color of each *ρ*(*v*) represents the number of the month analyzed, starting from May 2022 and ending in May 2023 (the values of the 13 months are codified in the colorbar; in particular, May 2022 corresponds to 1 and May 2023 to 13).

The results in Fig 2 show that the distributions *ρ*(*v*) are associated to completely discrete, half-discrete, or continuous values of *v*. For example, some distributions contain completely discrete values, which is the case of systems in Hartford (c) and Nashville (g) with relative maximums in specific values of *v*. This situation may be due to limitations in the GPS devices on the vehicles that only record specific values of velocity. Also, there are systems with half-discrete distributions, this is, with a combination of data that include discrete and continuous values of the velocities. This is the situation for the systems in Austin (a), Brampton (b), Louisville (d), Maui (e), and San Antonio (i). Moreover, the results show two cases with continuous records of *v*: Mexico City (f) and Rio de Janeiro (h), the two largest systems considered in our study. In addition, it is observed that every month, the distributions *ρ*(*v*) seem similar. This feature is maintained no matter the temporal window considered, weekly or daily, showing that statistically the global vehicular activity of bus rapid transit systems remains with no major changes through time. Finally, it is observed in the curves for *ρ*(*v*) a relative maximum of frequencies that lies in the range 20 km/h < *v* < 60 km/h, after which the values of probability quickly decline until the records of *v* > 80 km/h are very low. This result is explained by the speed limits of cities and policies of security on public transportation systems.

### Local activity of vehicles: From data to similarity networks

In the previous section, we explored the probability distributions *ρ*(*v*) of velocity at the global level in each system; the results show no major differences over time. In this section, we explore the data at the local level, partitioning all the systems into polygons or segments and analyzing the records within each of them. Those segments are described by an elongated rectangle defining geographical zones delimited by two consecutive stations in the same line with no cross with another one. In more complex cases we define general polygons that consist of the fusion of several overlapped segments. The segmentation of a BRT system allows us to analyze the vehicular activity at the local level to have a finer resolution of the properties, which gives detailed knowledge of vehicular activity.

In this way, if a BRT system is divided into N segments, the datasets of vehicular activity allows to generate the set of probability densities *ρ*_*i*_(*v*) of the velocity *v* at local level for each segment i=1,2,…,N. Once we obtained the distributions *ρ*_*i*_(*v*) for a specific period of time, the results can be compared using the Kullback-Leibler divergence $D_{\text{KL}}\left(i,j\right)$ to measure the similitude between a pair of probability densities *ρ*_*i*_(*v*) and *ρ*_*j*_(*v*), defined as [11, 50]

$$\begin{array}{c} D_{\text{KL}}\left(i,j\right)\equiv \int_{0}^{v_{\text{max}}}\rho_{i}\left(v\right)\;\text{log}\;\frac{\rho_{i}\left(v\right)}{\rho_{j}\left(v\right)}dv, \end{array}$$ (4)

where *v*_max_ = 90 km/h is the maximum speed considered in the analysis.

By definition $D_{\text{KL}}\left(i,j\right)$ is not equivalent to $D_{\text{KL}}\left(j,i\right)$; in this manner, it is useful to consider the symmetric measure [11]

$$\begin{array}{c} D_{\text{KLS}}\left(i,j\right)\equiv \frac{1}{2}[D_{\text{KL}}\left(i,j\right)+D_{\text{KL}}\left(j,i\right)]. \end{array}$$ (5)

Then, through the use of $D_{\text{KLS}}\left(i,j\right)$ in Eq (5), all the values generated from the comparison of the vehicular activity for all the segments i,j=1,2,…,N are consigned in a symmetric matrix with elements $D_{\text{KLS}}\left(i,j\right)$. In particular, if $D_{\text{KLS}}\left(i,j\right)\approx 0$, the vehicular activity between segments *i* and *j* is similar and, the opposite case is represented by a large value. Hence, we refer to the array with elements $D_{\text{KLS}}\left(i,j\right)$ as *similarity matrix*, because it quantifies the similarity between the vehicular activity in all the polygons in a BRT system.

Furthermore, it is possible to apply the methods of network science to analyze the similarity matrix of each BRT system. To this end, we define a network of size N in which nodes represent segments and edges the similarity in the movement of vehicles. In this way, two nodes *i*, *j* are connected if the respective segments have similar probability densities *ρ*_*i*_(*v*) and *ρ*_*j*_(*v*). To generate this structure, it is necessary to define what is considered sufficiently similar using a threshold value *H*. If two systems have values $D_{\text{KLS}}$ in Eq (5) lower or equal than *H* then these segments are considered similar. All this information defines a similarity network for each value *H*. The respective N×N adjacency matrix of the network is denoted as **A**(*H*), with elements *i*, *j* given by [11, 13]

$$\begin{array}{c} A_{ij}\left(H\right)=\{\begin{array}{cc} 1\; & D_{\text{KLS}}\left(i,j\right)\leq H,\\ 0 & \text{otherwise}. \end{array} \end{array}$$ (6)

Additionally, we require *A*_*ii*_(*H*) = 0 for *i* = 1, 2, …, *N*, to avoid self loops. From the symmetry of the distance $D_{\text{KLS}}$, follows the symmetry of **A**(*H*), defining an undirected network.

**Fig 3:**
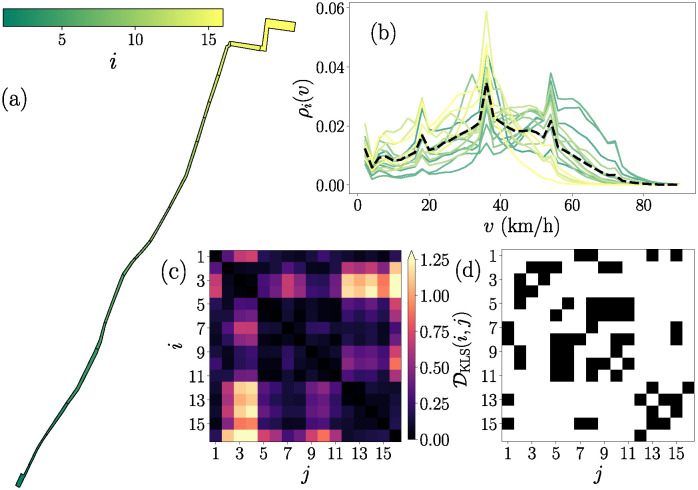
Vehicular activity at the local level for the BRT system in Louisville. (a) Segmentation of the system with N=16 polygons. (b) Probability density *ρ*_*i*_(*v*) for each polygon i=1,2,…,N considering all the velocity records of vehicles in the interval 0 km/h < *v* ≤ 90 km/h in each segment (bin counts are obtained using the size Δ*v* = 2 km/h). The dashed curve represents the probability density *ρ*(*v*) for the entire system. (c) Similarity matrix formed with the elements $D_{\text{KLS}}\left(i,j\right)$ for all pairs of segments i,j=1,2,…,N, the values of the elements are encoded in the colorbar. (d) Adjacency matrix **A**(*H*) describing a similarity network obtained after applying a threshold value *H* = 0.1 on (c), binary entries are codified in white for 0 and black for 1.

In Fig 3 we illustrate the ideas and concepts presented in this section. We consider the simplest BRT system studied which is located in the city of Louisville, Kentucky. In Fig 3(a) we show the segmentation of this system into N=16 polygons, each of them colored with the scale shown in the upper colorbar. The indexation of the polygons corresponds to the localization from south to north of their respective geographical centers. Here, it is possible to see the unique line in the city of Louisville. In Fig 3(b) we plot the corresponding probability density of velocities *ρ*_*i*_(*v*) for each polygon *i* for the entire dataset. The values are obtained using bins with size Δ*v* = 2km/h. The color of each distribution *ρ*_*i*_(*v*) corresponds to the color map shown in panel (a). In this image, we can see some characteristics of the different zones of the systems, for example, those whose activity differs more with respect to the mean distribution of the system, which is plotted with a dashed line, and corresponds to the analysis of the entire dataset. In contrast with the global analysis shown in Fig 2(d), the differences between the probability densities *ρ*_*i*_(*v*) are more notorious at the local scale.

In Fig 3(c) we depict the similarity matrix with dimension N×N, where N=16 is the number of polygons in the BRT system in the city of Louisville. The elements are calculated as $D_{\text{KLS}}\left(i,j\right)$, hence, we obtain a symmetric matrix and their values are represented by the respective colorbar. In Fig 3(d), we show the adjacency matrix **A**(*H*) obtained from the values $D_{\text{KLS}}\left(i,j\right)$ using the threshold value *H* = 0.1. The results shown in Fig 3 illustrate the methodology that can be implemented to express as a similarity network the massive dataset with geographic coordinates of vehicles in a BRT system and their velocity information. We use the parameter *H* as a threshold to transform the similarity matrices into undirected networks establishing a criterion for determining whether the vehicular activity within two given segments could be considered similar or not. The study of all the possible similarity networks generated by varying *H*, from isolated nodes to a fully connected graph, allows the identification of similarities at different scales using community detection algorithms; these results are presented in the next section for the nine BRT systems considered in this study.

## Results and discussion

### Similarity matrices

**Fig 4:**
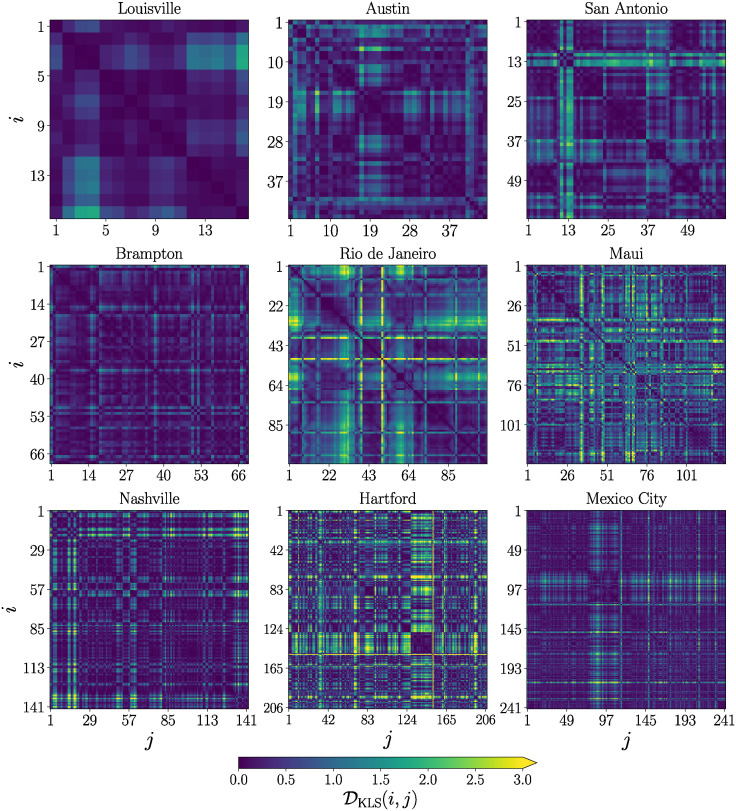
Similarity matrices for nine BRT systems. Matrices are sorted by using the number of segments N considered in each system. The value of each entry $D_{\text{KLS}}\left(i,j\right)$ is obtained using Eq (5) and encoded in the colorbar.

Once we discussed the general methods to map the data of the movement of vehicles to a network, in this section we apply these tools to analyze the datasets from our nine BRT systems. This approach allows us to identify properties common to this type of transportation system and features that characterize each system individually. In Fig 4, we present the similarity matrices for all systems, sorted by their respective number of segments (polygons) N; this is the number of nodes of the similarity network. We have N=16 for Louisville, N=45 for Austin, N=60 for San Antonio, N=69 for Brampton, N=103 for Rio de Janeiro, N=125 for Maui, N=142 for Nashville, N=206 for Hartford and N=242 for Mexico City. The respective N×N similarity matrices are obtained through the comparison of the vehicular activity in each segment, with values $D_{\text{KLS}}\left(i,j\right)$ obtained using Eq (5) and codified in the colorbar. Using $D_{\text{KLS}}\left(i,j\right)$, we can apply a threshold value *H* to transform similarity matrices into adjacency matrices and, therefore, to obtain networks associated to each system. In the following, we apply different network science methods to identify properties of collective patterns that arise at different scales in BRT systems.

#### Properties of similarity networks describing BRT systems

A central concept in network theory is the connected component. In particular, an undirected network is considered connected if there is a path between every pair of nodes *i*, *j*; otherwise, it is classified as a disconnected network. In a disconnected network, two or more connected components exist; these are connected subgraphs of the network [21]. In complex systems represented as networks, this concept helps to identify groups of elements with no interaction or relation to other groups. Furthermore, examining the relationship between the sizes of connected components is crucial, as it relates to percolation in networks [30, 51] and, consequently, to phase transitions in the system [30, 47, 51, 52]. Percolation can be observed in networks where the number of links grows in direct relation to a certain parameter. A specific case of connected components is the giant component, also known as the Largest Connected Component (LCC). A criterion for determining the percolation point involves comparing the sizes of the LCC and the Second Largest Connected Component (SLCC). Initially, both connected components grow together until the SLCC reaches its maximum size. Subsequently, the SLCC decreases rapidly, while the LCC undergoes a sudden growth, leading to an explosion of connectivity. At this critical point, percolation occurs.

**Fig 5:**
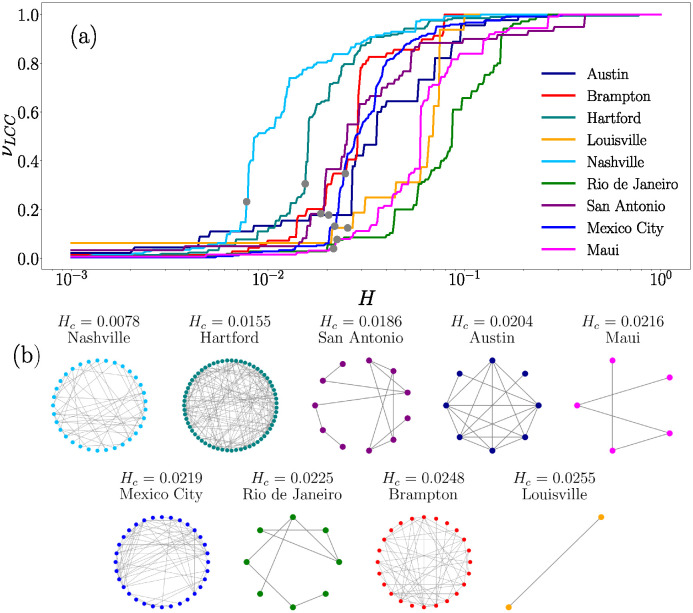
LCC size of similarity networks for different values of *H*. (a) Fraction of nodes *ν*_*LCC*_ in the LCC as a function of the value *H* for the nine BRT systems. The dots indicate the value *H*_*c*_ where percolation is detected. (b) LCC for the value *H*_*c*_ indicated in each panel (the systems are sorted according to the values of *H*_*c*_).

We search for connected components in the networks associated with vehicular movement to identify groups of polygons where activity can be considered similar. Particularly, we focus on the LCC. In Fig 5 we show some features of all the LCC for the respective value of *H* in the range [0, 1]. In Fig 5(a) we plot the fraction of nodes *ν*_*LCC*_ in the LCC with respect to the total number of segments N, the values obtained are presented as a function of *H*. The results exhibit an interval of values *H* with a rapid growth of the *ν*_*LCC*_.

The abrupt change in the characteristics of the systems is closely associated with a phase transition, and, in networks, this behavior is characteristic of percolation limits. In Fig 5(a) we highlight with dots the value *H*_*c*_ where the percolation occurs for each network. This result is obtained by comparing the sizes of the LCC and the SLCC. Notably, the Nashville system exhibits the fastest growth in similarity, indicating that for a low chosen *H* value, a significant portion of the system displays similar vehicular activity. In Fig 5(b) we show the graphs for the networks when the percolation point is reached, the systems are sorted using corresponding threshold *H*_*c*_. The color of the nodes corresponds to the color of the curves in Fig 5(a).

#### Community structure methods

Once we defined a network, it is possible to identify groups of nodes highly connected among themselves and poorly connected with nodes outside. This structure is crucial because it shows similar characteristics in a group of elements in the system represented by a network. This feature is commonly referred to as a *community structure*, and it constitutes a fundamental topic in network science [53]. Over the last two decades, this field has proven successful in studying various complex systems [54]. Different algorithms have been developed to detect communities, addressing various types of networks. Nevertheless, the definition of community is quite vague, and this is one of the reasons for the variety of methods to detect them [21]. Some of the most popular algorithms are those based on modularity [53].

**Fig 6:**
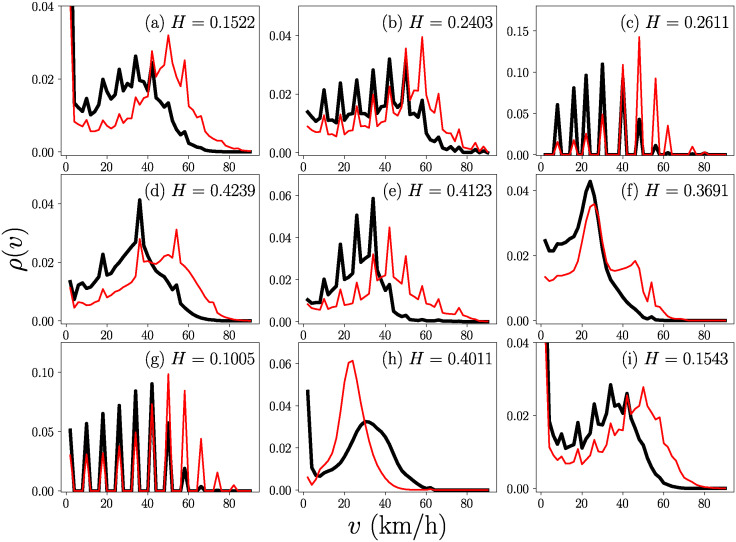
Vehicular activity in a classification with two communities. Probability density *ρ*(*v*) for segmentation of the systems in: (a) Austin, (b) Brampton, (c) Hartford, (d) Louisville, (e) Maui, (f) Mexico City, (g) Nashville, (h) Rio de Janeiro and (i) San Antonio, considering *v* the range 0 < *v* ≤ 90 km/h with a bin size Δ*v* = 2 km/h. The results for $C_{1}$ are presented in black and for $C_{2}$ in red. The threshold *H* is indicated in each panel.

In the following, we explore the community structure in all the LCCs to know if there are groups of polygons with similar vehicular activity. We use the Clauset-Newman-Moore greedy modularity maximization [55], implemented in the library networkx in Python [56]. In Fig 6, we plot the total probability density of velocities of the two communities found within the LCC for the *H* value indicated in each plot. The panels represent cities in alphabetical order: (a) Austin, (b) Brampton, (c) Hartford, (d) Louisville, (e) Maui, (f) Mexico City, (g) Nashville, (h) Rio de Janeiro, and (i) San Antonio. The results consider velocities in the range 0 < *v* ≤ 90 km/h and a bin size Δ*v* = 2km/h. The main feature observed in Fig 6 across all systems is the presence of a low-velocity community (denoted as $C_{1}$ and represented with black thick continuous lines) and a high-velocity community (denoted as $C_{2}$ and represented with red thin lines). Thus, using the threshold *H*, it is possible to categorize polygons based on their velocities, exclusively utilizing the data generated by the vehicles in BRT systems. A similar analysis was performed using a threshold *H* that generates three communities.

The properties of the analysis with two and three communities are presented in Table 3. In this table are reported the names of the cities in alphabetical order, the value *H* used to form two communities $C_{1}$ and $C_{2}$; and the value *H* considered to form three communities $C_{1}$, $C_{2}$ and $C_{3}$. The table also presents the number of segments that form each community as well as the the values of 〈*k*〉 (average number of segments with similar vehicular activity of a given one in the same community), the average shortest path 〈*l*〉 describing the global connectivity of the community and the global clustering coefficient 〈*C*〉. The last two columns report characteristics of vehicular activity within the group of segments in each community: the average velocity 〈*v*〉 and the standard deviation *σ*_*v*_.

**Table 3:** Properties of the communities in the LCC. The presented features correspond to the cases where two ($C_{1}$, $C_{2}$) and three communities ($C_{1}$, $C_{2}$, $C_{3}$) are formed. In the first column are presented the names of the cities analyzed. Then, we show the value *H* used to generate the network, the communities detected, and the number of segments in each group. The next columns show the average degree 〈*k*〉, the average shortest path 〈*l*〉, and the average clustering 〈*C*〉. The last two columns include the average velocity 〈*v*〉 of vehicles in the entire community and their respective standard deviation *σ*_*v*_.

City	*H*	Community	Segments	〈*k*〉	〈*l*〉	〈*C*〉	〈*v*〉 (km/h)	*σ*_*v*_ (km/h)
Austin	0.1522	$C_{1}$	25	10.72	1.7	0.74	24.19	17.18
		$C_{2}$	19	9.68	1.87	0.71	38.64	21.14
	0.1019	$C_{1}$	6	2.33	1.67	0.61	23.7	19.63
		$C_{2}$	19	8.53	1.76	0.76	24.75	16.22
		$C_{3}$	18	7.0	2.14	0.59	38.77	21.28
Brampton	0.2403	$C_{1}$	38	26.42	1.31	0.88	32.07	17.68
		$C_{2}$	31	25.48	1.15	0.92	42.99	19.68
	0.0554	$C_{1}$	22	9.82	1.79	0.79	33.08	18.87
		$C_{2}$	13	4.62	2.13	0.8	35.8	16.21
		$C_{3}$	24	9.5	1.79	0.72	42.19	19.59
Hartford	0.2611	$C_{1}$	103	53.5	1.54	0.8	27.87	16.37
		$C_{2}$	103	55.57	1.57	0.79	45.71	17.91
	0.1092	$C_{1}$	101	25.64	2.29	0.69	26.43	15.11
		$C_{2}$	97	29.98	2.02	0.72	41.57	17.42
		$C_{3}$	6	3.33	1.33	0.69	54.24	11.78
Louisville	0.4293	$C_{1}$	8	7.0	1.0	1.0	29.05	14.06
		$C_{2}$	8	7.0	1.0	1.0	41.1	17.52
Maui	0.4123	$C_{1}$	61	31.93	1.54	0.77	22.72	12.91
		$C_{2}$	64	30.97	1.77	0.81	42.86	21.06
	0.2212	$C_{1}$	59	17.8	2.03	0.7	22.57	12.86
		$C_{2}$	49	24.2	1.55	0.75	33.12	15.05
		$C_{3}$	10	3.8	1.82	0.65	43.67	20.65
Mexico City	0.3691	$C_{1}$	110	93.45	1.15	0.93	20.13	11.13
		$C_{2}$	132	102.77	1.25	0.9	27.41	14.26
	0.0762	$C_{1}$	99	20.77	2.33	0.64	20.07	10.84
		$C_{2}$	86	24.74	1.89	0.66	24.73	13.28
		$C_{3}$	45	7.11	2.94	0.6	29.49	14.25
Nashville	0.1005	$C_{1}$	67	33.52	1.62	0.79	25.95	16.33
		$C_{2}$	74	32.08	1.9	0.79	40.5	19.5
	0.0414	$C_{1}$	37	10.22	2.22	0.63	24.83	15.04
		$C_{2}$	45	20.98	1.69	0.78	32.04	17.08
		$C_{3}$	49	14.94	2.06	0.7	40.75	18.56
Rio de Janeiro	0.4011	$C_{1}$	51	18.9	1.94	0.72	20.91	15.48
		$C_{2}$	54	40.19	1.28	0.88	22.33	7.6
	0.237	$C_{1}$	12	3.67	2.11	0.66	13.62	13.04
		$C_{2}$	52	24.77	1.6	0.75	22.23	7.55
		$C_{3}$	40	9.65	2.21	0.62	30.31	13.01
San Antonio	0.1543	$C_{1}$	30	14.53	1.67	0.79	25.56	16.82
		$C_{2}$	25	15.28	1.42	0.79	35.14	20.44
	0.0912	$C_{1}$	22	7.27	2.06	0.76	24.38	17.13
		$C_{2}$	11	8.73	1.13	0.92	32.85	15.77
		$C_{3}$	20	9.7	1.59	0.79	36.56	20.1

Our findings also show that by reducing the value *H* (i.e., by increasing the requirement to consider two segments as similar), the nodes within the LCC reorganize into new communities. In particular, the values of 〈*v*〉 in Table 3 show a classification with two communities with low velocity $C_{1}$ and high velocity $C_{1}$; whereas the results with three communities reveal a classification that includes now a medium-velocity having $C_{1}$, $C_{2}$, $C_{3}$ for low, medium and high averages velocities, respectively. The notable exception to this classification is the system in Louisville, where segments are only organized into two communities. Moreover, the velocity classification is more pronounced in the systems of Hartford, Maui, and Rio de Janeiro, where the velocity ratio between 〈*v*〉 for $C_{1}$ and $C_{3}$ is almost the double. The value of *H* used for the segmentation of the system into three communities generates groups with a certain balance in their respective sizes, but we observe that some systems have two strong communities that gather the majority of nodes. This behavior is present in Hartford and Maui, where the LCC has a community with a reduced number of nodes in comparison with the nodes in the two larger communities. In addition, turning into the network properties associated with each community. In general, we can say that these results are useful to gain some insights about the properties of similarity in vehicular activity. For example, we highlight the values of Louisville, where we obtain two fully connected graphs, then, for this specific *H* all the segments in the same community have similar vehicular activity. Regarding the other systems the magnitudes of 〈*k*〉 and 〈*C*〉 decrease with *H*; in contrast, 〈*l*〉 increases when the value *H* is reduced.

The results in this section show that the method implemented based on the representation of geolocalized information of vehicles in transportation systems as a similarity network of geographical zones allows the classification of the information at different scales depending on the degree of similarity controlled by the threshold limit *H*. The approach is general and applicable to similar information of diverse transportation modes (for example taxis, bicycles, metro) and is not limited to the analysis of velocities; since additional information like the state of traffic can be incorporated in the definition of the similarity. In the particular case of BRT systems, we can conclude that the method implemented is effective in classifying segments based on velocities and positions of vehicles.

## Conclusions

We study bus rapid transit BRT systems located across the Americas, focusing on two aspects: infrastructure and vehicular activity. In the first part of the research, we adapted a method used to study connectivity in metro systems [23] to explore properties of BRT systems. Our findings show that the infrastructure of BRT systems exhibits unique characteristics that contrast with other complex networks observed in human mobility such as taxi services, bike-sharing systems, or airports. In the second part, we analyze millions of records of active vehicles in BRT systems including velocity and position (latitude and longitude). We explore vehicular activity across the entire system to outline activity patterns. Furthermore, we refine our analysis by examining the movement of vehicles dividing the systems into segments (polygons). We compare the distribution of velocities within each system, identifying differences using the symmetrized Kullback-Liebler divergence to the probability densities of velocity records for every pair of polygons. These results are organized as a similarity matrix for each system. We apply a threshold value *H* to transform the similarity matrices into undirected networks. This approach allows us to establish a criterion for determining whether the vehicular activity within two given segments could be considered similar or not.

After obtaining the adjacency matrices describing similarity networks, we proceed to study the properties of vehicular activity through its network representation. Initially, we analyze the size of the LCC for different values of the threshold value *H*. We apply community detection algorithms to the LCC to identify similarities among the polygons. Our focus lies on analyzing cases where two or three communities are formed. As a result, we conclude that we can classify the polygons of the systems in groups characterized by velocities: high and low velocity for a classification with two communities; and high, medium, and low velocity for a segmentation of the BRT systems considering three communities. Additionally, we examine the network properties of each community to characterize the connectivity within them. For instance, by quantifying the average degree in the graph of each community, we can infer the average number of polygons that exhibit similar vehicular activity within a given community. The study of the similarity networks generated by varying *H*, from isolated nodes to a fully connected graph, allows the identification of patterns at different scales using community detection algorithms.

Our findings show that the methods of network science explored are a useful tool to characterize at different scales BRT systems using geolocalized records of vehicular activity. The study is focused on nine BRT systems in the Americas, where detailed records of vehicular activity are available. Expanding the scope to include systems from other continents could provide a more comprehensive understanding of the activity patterns of vehicles in BRT systems. The methods implemented lead to the unsupervised detection of regions with similar characteristics considering the velocities and positions of vehicles in BRT systems. Other studies can incorporate the statistical analysis of different quantities of interest; for example, schedule adherence of the vehicles, carbon emissions, or user’s accessibility to stations. The classification of this information at different temporal scales is useful to deepen the understanding of urban transportation systems and, in collaboration with other professionals like transit specialists and urban planners, to draw up practical improvements introduced to optimize operational aspects like efficiency and connectivity at the scale of specific regions or the entire system. This approach is general and can be used in the study of other public transportation systems and pave the way for useful applications in the scientific study of urban areas.
